# The Role of Reactive Oxygen Species in Lung Cancer Development: Nanomedicine as a Therapeutic Strategy

**DOI:** 10.3390/biom15091316

**Published:** 2025-09-13

**Authors:** Manuel Olazábal-Morán, Elena Pérez, Adrián Esteban-Arranz, Antonio Garrido

**Affiliations:** 1 Nanocaging Research Group, Department of Biosciences, School of Biomedical and Health Sciences, Universidad Europea de Madrid, Villaviciosa de Odón, 28670 Madrid, Spain; elena.perez2@universidadeuropea.es; 2 Nanocaging Research Group, Department of Pharmacy and Nutrition, School of Biomedical and Health Sciences, Universidad Europea de Madrid, Villaviciosa de Odón, 28670 Madrid, Spain; adrian.esteban@universidadeuropea.es

**Keywords:** nano PROTACs, ROS, lung cancer, NRF2, targeted therapy, nanomedicine

## Abstract

Lung cancer remains a leading cause of mortality worldwide, driven by increased tobacco use, industrialization, and air pollution. Despite advancements in diagnostics and treatments, effective therapies are still lacking. Reactive oxygen species (ROS) play a dual role in cancer development, regulating key signaling pathways and activating cell death pathways, making them a promising target for new drugs. Research shows that wild-type NRF2/KEAP1 lung tumors, which account for about 60% of lung malignancies, are sensitive to ROS induction, and mutated *EGFR1* lung tumors exhibit high ROS levels. Proteolysis-targeting chimeras (PROTACs) have emerged as a promising alternative to small molecule inhibitors (SMIs) for cancer treatment, addressing limitations like undruggability and drug resistance. However, these face challenges such as limited cell penetration and potential toxic side effects. Nanotechnology has introduced “nano-PROTACs,” enhancing tissue accumulation, membrane permeability, and controlled release. In this review, the keystones of ROS in lung cancer will be summarized. Also, a potential therapy for tumors with wild-type NRF2 involving the delivery of ROS inductor nano-PROTAC will be designed. This potential therapy could suppose a potential therapeutic strategy for lung cancer patients with these genetic characteristics.

## 1. Introduction

**Figure 1:**
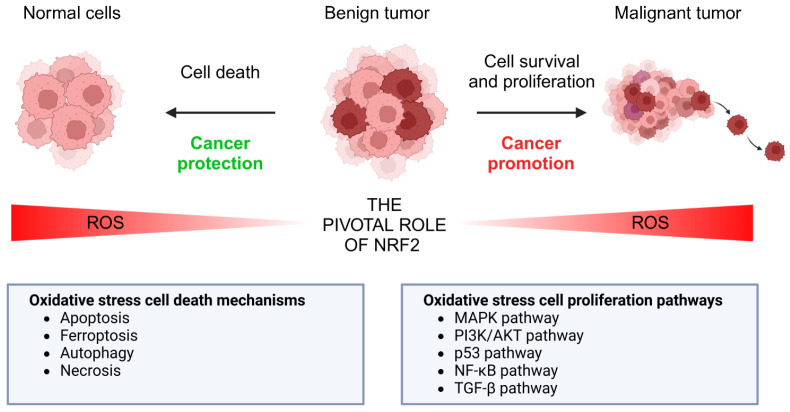
The pivotal role of ROS in tumorigenesis. In cancer, ROS regulate diverse signaling pathways like MAPK, PI3K/Akt, and NF-κB, promoting cell proliferation, survival, and metastasis. However, high ROS levels can cause the establishment of oxidative stress, leading to protein, lipid and DNA damage, cell cycle arrest, and apoptosis, potentially suppressing tumorigenesis. Thus, ROS act as both promoters and protectors from cancer, depending on their concentration and cellular context.

**Figure 2:**
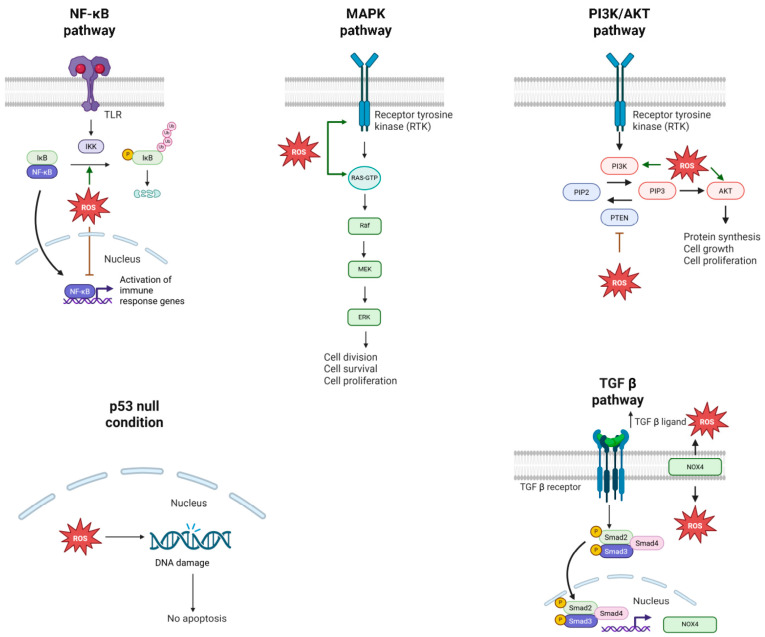
Signaling pathways involved in ROS promoting lung cancer. Reactive oxygen species (ROS) modulate key signaling pathways in lung cancer, including NF-κB, MAPK, PI3K/AKT, p53, and TGF-β. Each pathway shows context-dependent responses to oxidative stress, with ROS acting as both promoters and inhibitors of signal transduction. These interactions highlight the central role of ROS in tumor initiation, progression, and as potential therapeutic targets in lung cancer.

Cancer stands as a devastating illness and ranks among the primary causes of mortality globally. Lung cancer, in particular, accounts for the highest number of hospitalized patients and exhibits poor prognosis indicators along with extremely low survival rates, which consistently stem from these figures [1,2]. In fact, the survival rate for lung cancer significantly decreases depending on the stage of diagnosis. While 65% of patients diagnosed at stage 1 survive for five years or more, this rate drops to 15% at stage 3 and further declines to just 5% at stage 4 [3]. This dramatic trend appears to be attributed, at least partially, to the growing availability of tobacco, the industrialization of developing nations, and exposure to pervasive air pollution [4].

Lung cancer (LC) encompasses a diverse group of diseases, varying in both histological and molecular characteristics [5]. Histologically, LC is categorized into two primary groups: non-small cell lung carcinoma (NSCLC), which accounts for approximately 85% of LC cases, and small cell lung carcinoma (SCLC), comprising the remaining 15%. NSCLC is further subdivided into adenocarcinoma (LUAD), lung squamous cell carcinoma (LUSC), and large-cell carcinoma [5,6]. This classification system is based on the distinct cellular origins of each type. SCLC originates from neuroendocrine cells, while NSCLC subtypes have different cellular sources. Specifically, LUAD develops from alveolar type II (AT2) epithelial cells and/or cells within bronchioalveolar duct junctions. LUSC arises from stratified squamous epithelial cells and is not found in non-keratinizing epithelia. Large-cell carcinoma (LCC) stems from lung epithelial cells and represents a heterogeneous group of malignancies that lack the cyto-architectural features characteristic of LUAD and LUSC subtypes [6,7].

Significant scientific endeavors have focused on creating novel therapeutic approaches for LC, particularly LUAD and SCLC [8,9]. However, clinical research demonstrates the restricted effectiveness of currently available treatments. Despite recent advancements in diagnostic techniques, medical interventions, and surgical procedures, the prognosis for LC remains disappointingly poor [5,10]. Consequently, the complex clinical landscape of LC, coupled with the tumor’s aggressive nature, underscores the urgent need to discover and establish new therapeutic strategies. These innovative approaches could potentially be applied to patients, enhancing their survival rates and overall quality of life. Recent advancements in bioinformatics and large-scale next-generation sequencing studies have enabled researchers to identify key mutations in genes associated with the onset and progression of various cancers [11,12,13,14]. The Cancer Genome Atlas project, among others, has revealed the most common genetic alterations in LC, highlighting the diverse mutation profile characteristic of these tumor types. Notably, genes involved in the antioxidant response, such as *NFE2L2* (which encodes for NRF2, the primary transcription factor in the antioxidant response) and its negative regulator KEAP1, are frequently mutated in LC subtypes. These genetic alterations include activating mutations in *NFE2L2*, loss of *KEAP1* function, and changes in copy numbers [15,16]. This prevalence suggests that LC may be heavily reliant on the activation of this signaling pathway, potentially representing a vulnerability that could be exploited for new therapeutic approaches. In line with this, recent research has shown that LCs with wild-type *NFE2L2/KEAP1* are vulnerable to acute stress induction, even when exhibiting increased *NFE2L2/KEAP1* copy-number ratios. This finding indicates that wild-type *NFE2L2/KEAP1* lung tumors might be treatable using localized acute ROS as a novel targeted therapy [17]. Importantly, this acute oxidative stress induction did not affect normal lung cells. However, it is crucial to note that redox homeostasis plays a vital role in maintaining normal cellular functions [18,19], and non-specific induction of acute oxidative stress could have severe consequences for LC patients. For this reason, the way to produce this acute and local stress induction should be fine tuning controlled, being essential to employ specific mechanisms to produce this acute stress only in LC cells.

In recent decades, the development of proteolysis-targeting chimera (PROTAC) has provided a novel and alternative strategy that successfully overcomes the most important health problems of small molecule inhibitors (SMI). These later, although arose as one of the most effective strategies for cancer treatment, present several limitations. Among these, undruggability towards proteins lacking active sites or bearing unexposed active sites, off-target effects due to high drug concentration, target protein feedback accumulation and drug resistance as result of protein mutations [20,21,22] are the most important. Even so, PROTACs are still facing some bottlenecks and challenges that should be considered, such as the limited cell penetration, the intracellular delivery and “hook effect” as well as the potential toxic side effects [23]. Accordingly, the advent of nanotechnology has represented a promising avenue to surmount these challenges associated with conventional PROTACs. In fact, the second generation of PROTACs, also named “nano-PROTACs” holds the potential to enhance specific tissue accumulation, augment membrane permeability and enable controlled release [24,25,26]. Given the nature of lung cancer, this tumor type is particularly well-suited for nano-PROTAC therapy. Accordingly, LUAD is typically located in the outer regions of the lung, while NSCLC is found centrally near the bronchial tubes. These strategic locations offer an advantage for nano-PROTAC treatment, as their proximity to airways facilitates tissue accumulation and enables controlled release. Aerosol therapy provides direct drug delivery, faster onset, lower dosage, and a less invasive approach compared to intravenous and other delivery systems. This method can be effectively employed for nano-PROTACs in lung cancer treatment. Additionally, the respiratory system produces pulmonary mucus, a gel-like substance that can hinder the effectiveness of inhaled treatments. However, the development of aerosolized PROTACs could potentially overcome this challenge through direct delivery and enhanced penetration.

Considering all aspects mentioned above, we hypothesized that a nano-PROTAC capable of inducing an acute ROS induction specifically in the lung cancer tissue might be a potential strategy for these patients with wild type NRF2/KEAP1 signature. Therefore, the main goal of this review is to summarize the most important findings related to the generation of nano-PROTACs, focusing on those related to the induction of ROS. Considering these findings, we will design a ROS-inductor nano-PROTAC system as a novel targeted therapy for wild type NRF2/KEAP1 lung tumors. Also, the relevance of this signaling pathway and its relationship with others, which are related to the initiation and progression of this tumor type, will be revisited.

## 2. The Role of ROS in Cancer: Focus on Lung Malignancies

Reactive oxygen species (ROS) are highly reactive molecules which are byproducts from the metabolism of molecular oxygen (O_2_) generated during aerobic respiration. ROS have a great impact on cell function, acting as potent chemical cues that regulate diverse signaling pathways into the cell. They can modulate cell growth, cell death and cell differentiation, among others. Nevertheless, due to their great reactivity, ROS must be tightly regulated because their accumulation can trigger many harmful effects, damaging to different biomolecules such as lipids, proteins and DNA [27,28]. To counteract these negative consequences, cells developed different antioxidant mechanisms to detoxify ROS. However, when these mechanisms fail to decrease ROS levels, the cell is lead to an imbalance in redox status, where the production of ROS exceeds cell antioxidant defenses. A persistent imbalance redox status promotes a phenomenon known as oxidative stress [29]. Understanding oxidative stress is essential for characterizing the etiology of many human diseases such as Alzheimer, atherosclerosis, chronic obstructive pulmonary disease and cancer, including lung cancer [30]. This knowledge is key to developing successful therapies whose targets are the regulation nodes of redox balance.

### 2.1. The Origin of ROS in the Cell

There is a plethora of ROS that are important in cell physiology. They can be classified as the reactivity that they present in (1) non-radical molecules like hydrogen peroxide (H_2_O_2_), and (2) free radical molecules (characterized for having an additional unpaired electron) as superoxide anion (O_2_^−^) and hydroxyl groups (OH^−^). To mitigate the presence of O_2_^−^, which is highly oxidizing and an angular precursor of several ROS [31], cells have acquired superoxide dismutases (SODs). These enzymes catalyze the conversion of O_2_^−^ into H_2_O_2_, a less reactive but still potentially harmful ROS [32]. H_2_O_2_, while less damaging than superoxide, can further decompose into water and oxygen through catalase or peroxidase activity, thus protecting cells from oxidative stress and maintaining cellular function [33].

Mitochondria is the primary source of cellular ROS, cellular place where electron chain transport (ECT) occurs. ECT, localized in the inner membrane of the mitochondria, consists of a series of proteins complexes that transport electrons from reduced electron donors to the O_2_, while pumping H^+^ to the intermembrane space to generate an electrochemical gradient employed to obtain ATP through oxidative phosphorylation. In this process, electrons leak from the different complexes of the ECT, interacting with O_2_ and producing O_2_^−^ that is converted to H_2_O_2_ by the SODs of the intermembrane space [34,35,36,37]. H_2_O_2_ is also highly produced in peroxisomes by several oxidoreductases [38]. Moreover, peroxisomes and mitochondria are closely interconnected and both peroxisome biogenesis and peroxisomal β-oxidation increases mitochondrial oxidative stress in several tissues [39,40,41]. Another important source of ROS is NAPDH oxidases (NOX), a family of membrane proteins with the ability to transport electrons through the plasma membrane and to produce H_2_O_2_ and O_2_^−^ [42]. Minor sources of ROS are generated by the actions of other enzymes such as monoamine oxidase (MAO), cyclooxygenase cytochrome P450 (CYP) and xanthine oxidase (XO) [43].

Interestingly, ROS can also be exogenously produced by lifestyle factors, such as alcohol consumption, heavy metals ingestion, radiation, tobacco smoking and air contaminants exposition [44]. Among the different organs in the body, lungs are constantly exposed to chemical hazards due to is where gas exchange is produced, leading to both oxidative stress and inflammation [45]. Among all exogenous factors, one of the most well-known ROS inducers is tobacco. Tobacco smoke contains nitric oxide (NO), an oxidant molecule that interacts with O_2_^−^ and produces peroxynitrite (ONOO^−^), a reactive compound that damages proteins and DNA and is responsible, among other factors, for lung cancer development. People exposed to occupational hazards (xenobiotics, biological agents, air pollutants…) and with chronic pneumonic infections exhibit increased ROS levels in the lungs that have also been related with lung malignancies [46].

### 2.2. Pivotal Role of ROS in Lung Cancer

ROS are important players in the development of malignancies. Growing evidence suggests that ROS play a pivotal role in cancer, as they can both promote and protect from tumorigenesis. On the one hand, cancer cells are characterized by carrying more ROS than normal cells [47]. ROS are important players both in initiation and promotion of cancer as they induce the activation of oncogenes, inactivation of tumor suppressor genes and promotion genomic instability, all of them hallmarks of cancer [48]. On the other hand, the massive accumulation of ROS induces several pathways of cell death, highlighting an important antioncogenic role that can be exploited by targeted therapy (Figure 1).

#### 2.2.1. Role of NRF2 in Lung Cancer

Among the different molecular regulators of redox balance, NRF2 (Nuclear factor-erythroid-derived 2-related factor 2)-KEAP1 (Kelch-like ECH-associated protein 1) pathway is one of the most important cellular signaling mechanisms that governs the antioxidant response and cellular defense against oxidative stress [7]. KEAP1 is a cysteine-rich protein that functions as a key negative regulator of NRF2, a transcription factor that controls the gene expression involved in oxidative stress response, detoxification, and cellular repair. Under normal conditions (unstressed conditions), KEAP1 binds to NRF2 in cytoplasm, facilitating its ubiquitination by the Cul3-RBX1 E3 ligase complex, leading to NRF2 degradation by the proteasome [49,50]. Nonetheless, the cysteine-riche region of KEAP1 acts as a redox sensor and under high ROS levels these cysteines are oxidized [51,52,53]. The modifications of the cysteine residues disrupt NRF2-KEAP1 interaction, leading to NRF2 stabilization, accumulation and translocation to the nucleus to regulate a complex gene expression program. NRF2 binds to Antioxidant Response Elements (ARE) in the promoter region of a plethora of target genes such as genes mediators of the antioxidant response, anti-apoptotic proteins, cell cycle regulators, heme metabolizing proteins and xenobiotic metabolizing enzymes [54,55].

NRF2-KEAP1 pathway is usually altered in NSCLC tumors due to gain-of-function mutations of *NFE2L2* (which encodes for NRF2) or loss-of-function alterations of *KEAP1* [56]. Specifically, *NFE2L2* is more frequently mutated in LUSC (15%) than LUAD (3%) and *KEAP1* is usually altered both in LUSC (12%) and LUAD (17%) [57]. Interestingly, this pathway plays a pivotal role in lung cancer acting both as a protective mechanism in the early stages of carcinogenesis and as a promoter of cancer progression in established tumors. This duality depends largely on the stage of cancer, the microenvironment, and the molecular alterations in the pathway [58]. Experiments in animal models of lung cancer have been key to clarifying the differential function of NRF2. In LUAD models, pre-activation by sulforaphane prevents tumor initiation but promotes tumor growth once initiated [59]. *Nrf2*-deficient mice show increased tumor loci early after carcinogen exposure, but less malignancy later [60]. *Keap1* knockdown mice show reduced growth of induced tumors but higher tumorigenicity in transplanted *Keap1*^−/−^ cancer cells [61]. These findings suggest NRF2′s complex roles in cancer, with both protective and detrimental effects, especially in NSCLC.

Considering all this evidence supporting the role of NRF2/KEAP1 signaling pathway in lung cancer development, this opens NRF2 as a new therapeutic target to develop antineoplastic treatments for lung malignancies.

#### 2.2.2. Role of ROS in Promoting Lung Cancer

ROS are essential chemical cues that regulate cell signaling mainly by oxidation of cysteine and tyrosine residues in several proteins, modifying their conformation and protein–protein interactions [62,63]. Among proteins affected by ROS, there are several pivotal players in the most important molecular pathways involved in lung cancer initiation and promotion (Figure 2).

The nuclear factor-κB (NF-κB) signaling pathway consists of a panel of transcription factors (NF-κB) that were originally described as the essential modulators of the inflammatory response. However, this pathway is also important in cell proliferation, angiogenesis and metastasis [64]. NF-κB transcription factor is increased in SCLC and NSCLC, as initiates cancer by inhibiting apoptosis and promoting DNA damage [65]. Not only is important in lung cancer initiation but metastasis, as NF-κB induce the expression of metastasis-associated protein 2 (MTA2) in the lung [66]. This increase in NF-κB is also important in conferring tumoral cells resistance to chemotherapy [67]. The role of oxidative stress in NF-κB is complex and different depending on the pathological context. The accumulation of ROS tends to degrade IκBα, one of the main negative regulators of the pathway, and activate NF-κB [68,69]. However, ROS also diminish NF-κB signaling by the oxidation of specific cysteine residues of the subunits of the NF-κB that restrain DNA binding and by avoiding IκBα degradation in the proteasome [60,70]. The fine-tuning of antioxidant systems is also important in this pathway, as it has been evidenced that SOD2 is needed in lung cancer because it expresses IKKβ (an upstream activator of NF-κB) [71].

The Mitogen-activated-protein-kinase (MAPK) signaling is one of the essential nodes of regulation of cell division, cell survival, apoptosis, cell differentiation and proliferation [72]. This pathway is initiated by different membrane receptors that activate the small GTPase RAS. Once activated, RAS orchestrates the sequential activation of a cascade of serine-threonine kinases that modulate the function of different molecular players in the cell. A sustained activation of the pathway due to activating alterations of Kirsten rat sarcoma viral oncogene homolog (KRAS) (12–30%) and BRAF (4%) is frequently observed in lung cancer samples [73]. In fact, it has been recently reported that a new inhibitor of KRAS^G12C^, Sotorasib, is effective to treat locally or metastatic NSCLC [74]. The main effector regulated by ROS in this pathway is RAS because all isoforms have different cysteines in their sequence that are target of redox modifications. Moreover, the oncogenic mutations G12C and G13C in the isoform KRAS create new sites where ROS can modify the activity of the protein [75]. Mitochondrial ROS are essential for Kras-driven tumorigenicity in a mouse model of lung cancer [76]. MAPK pathway is also regulated by ROS in an upstream manner in pancreatic cancer, as oxidation of EGFR drives precancerous lesions [77].

The phosphatidylinositol 3-kinase (PI3K/AKT) pathway is a critical signaling cascade involved in cell growth, survival, metabolism, and migration. It begins with the activation of cell surface receptors, mainly receptor tyrosine kinases (RTKs), which activate the lipid-kinase activity of PI3K to produce PIP3 (Phosphatidylinositol (3,4,5) trisphosphate) in the plasma membrane, an important second messenger that activates downstream proteins like AKT [78]. PI3K pathway is one of the most frequently hyperactivated in lung cancer [79]. The two most common alterations in NSCLC are activating-mutations or increases in the number of *PIK3CA* (which encodes the p110α catalytic subunit) and reduction or elimination of PTEN expression, the negative regulator of this signaling cascade [80,81,82]. PI3K/AKT pathway and ROS are two sides of the same coin because hyperactivation of PI3K induces ROS production by alterations in mitochondrial metabolism and NOX activity [83]. Beyond that, ROS oxidize AKT in Cys 60 and Cys 70 and stabilize its binding to PIP_3_, maintaining PIP_3_ signaling [84]. PTEN is also inhibited by the oxidation of cysteine residues in the presence of H_2_O_2_ [85]. To avoid the great accumulation of ROS, PI3K/AKT pathway also promotes the expression of NRF2, one of the main antioxidant mechanisms of the cell.

p53 is a vital tumor suppressor protein that monitors various types of cellular damage, including DNA mutations, oxidative stress, hypoxia, and telomere shortening. Upon sensing such damage, p53 stabilizes and acts as a transcription factor that induces cell cycle arrest to repair the damage or apoptosis if the damage is irreversible [86]. Alterations of TP53 occur in more than 50% of human primary tumors [87]. Regarding lung cancer, mutations of *TP53* are common in LUAD (46%) and usually concomitant with *KRAS* alterations [88]. p53 is an important mediator of oxidative stress response as it induce the expression of genes that codify proteins important in the antioxidant response as tumor protein 53–induced nuclear protein 1 (TP53INP1), glutathione peroxidase (GPX) and aldehyde dehydrogenase (ALDH) [89,90,91]. On the other hand, under oxidative stress conditions, p53 inhibits the NRF2 antioxidant response to induce cell death by ROS accumulation [92]. This p53-mediated induction of ROS can be exploited to increase sensitivity in chemo-resistant types of NSCLC [93]. It has also been described that p53-mutant NSCLC are more sensitive to ROS induction as the antioxidant response mediated by p53 is altered [94]. ROS are also important mediators of p53 function, as its oxidation stabilizes the transcription factor [95]. Furthermore, NRF2 expression after ROS stimulation leads to NQO1 (NAD(P)H dehydrogenase [quinone] 1) generation, that interacts and stabilizes p53 in NSCLC [96,97].

Transforming growth factor (TGF)-β is a ligand that induces the activation of the SMAD transcription factors. These transcription factors regulate the expression of genes involved in differentiation, cell proliferation, survival, migration, and invasion [98]. TGF-β is overexpressed in NSCLC and it is a key signal to promote epithelial–mesenchymal transition (EMT) during lung tumor metastasis [99,100]. ROS have a dual role with TGF-β signaling as they are induced by this signal, but they also activate TGF-β. TGF-β upregulates NOX4 expression, leading to a ROS increase in lung cancer cells [101]. Moreover, TGF-β1 isoform suffers an active conformation change induced by ROS oxidation [102]. Even more, TGF-β1 levels are increased in the presence of ROS, leading to an EMT phenotype of lung epithelial cells and suggesting its potential action in lung cancer metastasis [103].

#### 2.2.3. Role of ROS in Protecting from Lung Cancer

As it has been previously indicated, ROS can directly damage different cellular structures and biomolecules, inducing cell death. The cell death pathways affected by ROS are extremely complex and depend on the cell origin and the microenvironmental context. The next paragraphs briefly summarize the main oxidative stress mediated cell death mechanisms.

ROS have the ability to induce apoptosis, a programmed cell death orchestrated by the sequential action of a family of cysteine-dependent aspartate proteases called caspases. After caspase effector activation, cells are destroyed and form apoptotic bodies that are engulfed by adjacent macrophages. There are two main apoptosis programs, the extrinsic and the intrinsic pathway. The extrinsic pathway is initiated by the binding of extracellular ligands (TNFα, FasL…) to the cognate receptors, which activate several caspases that regulate the assembly of death-inducing signaling complex (DISC), necessary to activate the effector caspases [104]. DISC formation is inhibited by the interaction with c-FLIP, whose levels are reduced in the presence of ROS because they target c-FLIP to be degraded in the proteasome [105]. The apoptotic intrinsic pathway is triggered by DNA damage, endoplasmic reticulum stress and oxidative stress because they activate pro-apoptotic factors of the BCL-2 family like BAX and BAK. Intrinsic apoptosis is mainly initiated by the liberation of cytochrome c from the mitochondrial membrane and its interaction with APAF-1 to generate the apoptosome, that activates caspase 3 and 9 [104]. As has been previously mentioned, a great bulk of ROS is produced in mitochondria. Their accumulation in this organelle promotes membrane permeabilization and the promotion of cell death [106].

Ferroptosis is an iron-dependent form of cell death characterized by the accumulation of ROS and lipid peroxidation. It involves mitochondrial shrinkage, membrane rupture, and depletion of glutathione, with disrupted antioxidant systems. The main morphological difference with apoptosis is that the nucleus and the chromatin condensations are not altered in ferroptosis [107,108]. Ferroptosis remains a mechanism of cell death that we know little about today, but it has been shown to be caused by lipid peroxidation. Depletion of glutathione (GSH) and glutathione peroxidase 4 (GPX4) promotes the oxidation of polyunsaturated fatty acid (PUFA) and the accumulation of lipid ROS that leads to ferroptosis [109].

Another type of programmed cell-death related to ROS is autophagy, where cells break down and recycle damaged components. It has been established that ROS induce autophagy in cancer cells, and it can be potentially used as therapy. This induction occurs because ROS inhibit mammalian target of rapamycin 1 (mTORC1), the main negative regulator of autophagy, by inactivating autophagy-related gene-4 (ATG4) and AMP-activated protein kinase (AMPK) [110,111]. In fact, administration of rapamycin with inhibitors of HSP90 in RAS-dependent lung tumors promotes mitochondrial damage and oxidative stress and tumor growth reduction [112].

Necrosis has been usually defined as the main pathological type of cell death, but several studies suggest that it can be regulated by a diverse panel of proteins forming another programmed cell death knows as necroptosis [113]. Necroptosis works in a similar way to the extrinsic apoptosis, as it is initiated by the simulation of membrane receptors (FasR, TNFR…) by soluble ligands that interact and activate receptor-interacting protein kinases (RIPs). The activation of RIP3 increases ROS production in the mitochondria and enhances this type of cell death [114].

Considering all these findings, ROS play a pivotal role in lung cancer, promoting tumor growth by activating oncogenes, inducing genomic instability and promoting metastasis, while also triggering cell death through apoptosis, ferroptosis, necrosis and autophagy. These opposite effects complicate cancer progression but also open up therapeutic opportunities, where targeting ROS-induced cell death could be a promising strategy to treat lung cancer and overcome resistance to conventional therapies.

## 3. Targeted Therapy for Lung Cancer

### 3.1. The Origin of Targeted Therapy: Introduction to Small Molecule Inhibitors

Cancer therapy has relied on chemotherapy for a very long period, consisting of this approach in the use of a broad-spectrum of non-specific cytotoxic drugs characterized for having toxicity and adverse secondary effects. Thanks to the better comprehension of cancer biology that we have today, a shift has occurred into the development of new targeted cancer therapies [115]. The principal difference in the former in comparison with traditional chemotherapy is that these drugs specifically kill tumoral cells and spare normal cells, diminishing the toxicity. Among the drugs used in targeted therapy highlight SMI because they have great pharmacokinetic properties and logistical advantages like easy storage and production. In fact, there are more than 80 SMI already being used to treat many different cancer types in different countries around the world [116]. In this context, the vast majority of the developed SMI target kinases that orchestrate different signaling pathways involved in cell division, cell proliferation and cell survival. Nevertheless, modulation of ROS levels in cancer cells is a promising angle of targeted therapy that has not been deeply considered. In fact, the previously commented pivotal role of ROS in cancer allows different targeted therapeutic strategies. From one perspective, ROS are essential triggers of cell transformation because they activate several pathways involved in cell proliferation. In this regard, administration of antioxidant drugs seems to be a plausible therapeutic scenario, which has been previously reported with the administration of antioxidant vitamins in patients with cancer, including lung malignancies [117,118]. However, recent investigations have proved that antioxidant compounds lead to a more rapid lung cancer progression [119], consequence of the opposite role of ROS in cancer development. On the other hand, ROS are cell death inducers that can be exploited to target tumoral cells [120]. This complex role of ROS in tumorigenesis seems to deeply rely on tumor stage and tumor microenvironment, which is an essential piece to generate ROS. Consequently, the rational design of efficient ROS-targeted therapies needs to take into consideration both the stage of the tumor and the physicochemical and biological conditions of the tumor microenvironment.

Even though targeted therapy has been key in the development of efficient antitumoral therapies, there are two disadvantages that need to be faced. The first one is that after clinical use of SMIs tumoral cells acquire drug resistance by different molecular mechanisms [121,122]. One of these resistant mechanisms occurs due to genomic instability of tumoral cells because it induces mutations and amplifications of genes that confer resistance to the drug. For example, the amplification of MET in NSCLC is key to developing resistance to EGFR inhibitors [123]. Another important issue in relation to drug resistance is Cancer Stem Cells (CSCs), cells present in tumors with stem cell properties that have been associated with the development of drug resistance [124]. Other resistant mechanisms include the expression of efflux transporters and deregulation of autophagy [125,126]. Another important drawback with targeted therapy is that there are some potential targets that are considered undruggable because their molecular structure lacks pockets for drug interaction. In this regard, the paradigmatic case of an undruggable target is KRAS, which has been undruggable after decades of research [127]. For this reason, it is imperative to enhance investigative efforts and the development of novel therapeutic strategies to increase treatment efficacy.

There are several approaches to develop new targeted therapies facing these main problems of SMI. Among them, one that is causing great expectation due to its potentiality, the use of Protein Degrader systems which would prevent drug resistance and undrugability. These systems consist of using molecules that target a specific protein to be degraded by the intracellular protein-degradation machinery, being PROTAC the spotlight of this technology.

### 3.2. The Revolution of Targeted Therapy: PROTAC Technology

Targeted protein degradation consists of modulating the ubiquitin-dependent proteolysis that occurs physiologically in the cell. This proteolysis mechanism is mediated by tagging damaged or non-required proteins with ubiquitin to send them to be degraded in the proteasome [128]. Ubiquitin is a protein that is attached to lysine residues of proteins by an isopeptidic bond, acting as a post-translational modification (PTM). To understand targeted protein degradation is key to comprehending how ubiquitination occurs, a complex three-step enzymatic cascade. Briefly, at first ubiquitin is attached to an E1 activating enzyme by a thioester bond in an ATP-dependent manner. Secondly, ubiquitin is transferred to an E2 conjugating enzyme, which can directly conjugate ubiquitin to a lysine of a target protein mediated by an E3 ligase or ubiquitin is conjugated to an E3 ligase that directly ubiquitinate the target protein without the action of the E2. As ubiquitin possesses several lysine residues in its sequence it can also be ubiquitinated, leading to the formation of polyubiquitin chains in the target protein. The role of this PTM varies depending on the residues of the lysines that are ubiquitinated and the topology of the polyubiquitin chains [129], but K48 polyubiquitin is clearly defined as a signal that targets proteins to be degraded in the proteasome [130]. Interestingly, ubiquitination is a reversible modification and ubiquitin proteins are recycled due to the existence of deubiquitinating enzymes that remove them from target proteins [131].

**Figure 3:**
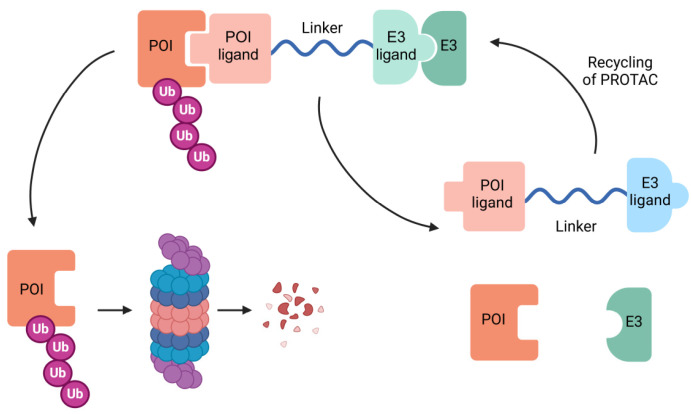
PROTAC structure. PROTAC molecules are bifunctional compounds that induce targeted degradation of specific proteins. They consist of two ligands: one binds to the protein of interest (POI), while the other recruits an E3 ubiquitin ligase. This interaction promotes ubiquitination and proteasomal degradation of the target protein. Once the POI is degraded, PROTAC is recycled and can interact again with the POI.

The first PROTAC was designed in 2001 by Deshaies and Crews [132]. This design consisted of using a protein-targeting chimeric molecule 1 (Protac-1) with the ability to recruit both the target protein to be degraded (methionine aminopeptidase-2) and the ubiquitin ligase Skp1-Cullin-F-box (SCF). Therefore, a PROTAC molecule is characterized by its heterobifunctional structure as it has one ligand that is attached to a protein-of-interest (POI) and another one that binds an E3 ligase, connected through a linker region (Figure 3). The design of these molecules is one of the critical steps in PROTAC technology. On the one hand, it is key to identify appropriate E3 ligases that effectively ubiquitinate the POI. More than 600 E3 ligases are encoded by the human genome, but only a few have shown potential in the development of targeted protein degradation [133]. Among these, research has focused attention on MDM2 (an essential regulator of p53 protein levels), IAPs (key players in caspases regulation during apoptosis), VHL (the E3 ligase that target HIF transcription factors to be degraded in the proteasome under normoxia) and cereblon (a E3 ligase with pleiotropic functions and essential in embryo development). Other E3 ligases that have been successful in PROTAC technology can be consulted in Table 1. On the other hand, the linker is crucial to establish a stable complex between POI and the E3 ligase. Usually, linkers are composed of polyethylene glycol and alkanes due to their flexible and soluble properties [134].

**Table 1:** E3 ligases under research for PROTAC technology.

E3 Ligase	Protein of Interest (POI)	Reference
**KEAP1**	**FAK** **Tau** **BRD4** **CDK9**	[135,136,137,138]
DCAF1	BRD4 FKBP12	[139]
**DCAF11**	**FKBP12** **AR**	[140]
DCAF15	BRD4 BRD7 BRD9	[141,142]
**DCAF16**	**PARP2** **FKBP12**	[143,144]
LM3BTL3	BRD2 FKBP12	[145,146]
**KLH20**	**BRD2** **BRD3**	[147]
AhR	BRD2 BRD3 BRD4	[148]
**FEM1B**	**BCR-ABL** **BRD4**	[149]
RNF4	BRD4	[149]
**RNF114**	**BCR-ABL** **BRD4**	[150]

Adapted table from Zhong et al., 2024 [151]. *KEAP1* Kelch-like ECH-associated protein 1, *FAK* focal adhesion kinase, *BRD* BET bromodomain protein, *CDK9* cyclin-dependent kinase 9, *DCAF* DDB1 and CUL4 associated factor, *KBP* FK506-binding protein, *AR* androgen receptor, *PARP2* Poly(ADP-ribose) polymerase 2, *L3MBTL3* Lethal(3)malignant brain tumor-like protein 3, *KLHL 20* Kelch-like protein 20, *AhR* aryl hydrocarbon receptor, *FEM1B* feminization 1 homolog B, *RNF* ring finger protein.

Nowadays, there are many ongoing clinical trials addressing the efficacy of targeted protein degradation to treat different human diseases, including cancer [151]. Even though hematological tumors are more accessible to these therapies, solid tumors are also under investigation, with NSCLC being deeply studied due to the high mortality associated with this malignancy. One of the most important driver mutations of NSCLC affects EGFR, an angular RTK that regulates epithelial cell signaling. Consequently, many SMIs have been developed to target this molecule, greatly improving NSCLC treatment [152,153,154]. Nevertheless, recurrency occurs after treatment because tumoral cells acquire resistance to these drugs, usually due to the EGFR T790M mutation [155]. All this knowledge has been applied to design PROTACs that specifically target mutant forms of EGFR to be degraded in the lysosomes [156]. These molecules have been proven to be effective to degrade EGFR both in cell lines and in tumors [157,158]. It has been recently published that orally administration of HJM-561 PROTAC is effective to diminish EGFR protein levels in patient-derived-xenograft models with EGFR triple mutants, which are common resistant forms of EGFR in patients with advanced NSCLC under Osimertinib treatment [159]. In fact, there are two clinical trials assessing how efficient HSK-40118 PROTAC is for targeting EGFR in NSCLC patients [160,161]. Beyond these clinical trials there are several promising preclinical studies addressing the efficacy of distinct PROTACs molecules with different targets in lung cancer, which have been recently reviewed by Li et al., 2023 [162].

While PROTACs represent a significant step forward in drug development by facilitating targeted protein degradation, they encounter several hurdles in clinical applications. Their distinct molecular structure often results in poor water solubility and limited ability to penetrate tissues [163]. Consequently, this poses challenges in drug formulation, absorption, and distribution, potentially necessitating higher doses and diminishing their therapeutic impact. Additionally, a significant issue with conventional PROTACs is their inability to target specific tissues. These compounds degrade proteins in both healthy and cancerous cells, which can lead to systemic toxicity [164]. Another critical challenge for traditional PROTACs is their metabolic instability. PROTACs can be quickly metabolized and eliminated from the body, reducing their therapeutic potential. Furthermore, due to their size and structure, PROTACs often face difficulties with oral bioavailability, complicating their effective administration through this route [165]. Therefore, it is essential to devise strategies to ensure PROTACs are effectively delivered to target tissues, and their integration with nanotechnology holds great promise in this regard.

### 3.3. Nanomedicine Avenues: Transforming Cancer Drug Delivery

Nanotechnology has become pivotal in the clinical application of PROTACs due to its unique physicochemical properties. The nanomedicine approach for drug delivery such as liposomes, nanoparticles and nanogels offer a diverse plethora of methods to load antineoplastic drugs [166,167,168,169]. These delivery systems are characterized by having optimal pharmacokinetic parameters that make them very useful in cancer therapy. By enhancing drug concentration at tumor sites, these systems can be precisely controlled in both space and time to interact specifically with target cells, and they can be combined with other antitumor therapies [24], addressing the key challenges previously noted with PROTACs. Incorporating PROTACs into nanoparticles or nanogels can significantly boost their solubility and permeability. These nanotechnology systems shield them from degradation and improve their ability to penetrate cell membranes [170]. Nano-PROTACs can be designed to target specific tissues or cells, thereby reducing off-target effects and minimizing systemic toxicity. This targeted delivery ensures that PROTACs reach the precise location where they are needed [170]. Additionally, encapsulating PROTACs in nanomaterials like nanoparticles or hydrogels can protect them from metabolic breakdown, enhancing their stability and extending their presence in the bloodstream. This increases their therapeutic effectiveness [170]. Consistent with this, nano-systems can be engineered to release PROTACs in a controlled fashion. This nanotechnology can respond to environmental changes such as pH and reduced glutathione modifications, which occur in tumor tissue, ensuring a sustained therapeutic effect over time and helping to maintain optimal drug levels in the treatment area. Furthermore, nano-formulations can enhance the bioavailability of PROTACs, making challenging administration routes, such as oral delivery, more feasible by improving their absorption in the gastrointestinal tract [170].

Nowadays, over 30 nanosystem types have entered clinical trials, highlighting their potential in modern medicine [171]. Among the different nano-systems to deliver PROTACs, nanoparticles (NPs) are currently under deep investigation because they can be used to deliver anti-neoplasic drugs to tumors poorly accessible by conventional treatments. There are different types of NPs which have potential delivery properties such as (i) lipid-based NPs, (ii) inorganic NPs and (iii) polymeric NPs.

Lipid-based NPs have emerged as a promising platform for delivering PROTACs, due to their excellent biocompatibility and flexibility. Lipid-based NPs are spherical vesicles composed of phospholipids monolayers or bilayers that cover an aqueous inner compartment, so it can deliver both hydrophilic and hydrophobic drugs [172]. Using phospholipids analogous to cellular plasma membranes enhances PROTAC intake by cells [173]. Nanoliposomes containing PROTACs targeting BRD4 have been useful to treat vemurafenib resistant melanoma cells in vitro [174]. However, PROTAC systems have low solubility in water, so using these NPs would require loading it into the lipid layer. It has been described that using a pre-fused PROTAC (E3 ligase is already attached to the linker region) delivered by lipid-based NPs greatly improves the degradation of the POI [175].

Inorganic NPs, such as gold and mesoporous silica NPs, are promising candidates for PROTAC drug delivery. Some of the advantages of this type of NPs are that they are easy to prepare, and they are rigid, which is useful to avoid leakage of the treatment during delivery. On the one hand, gold NPs have shown great potential to deliver PROTACs, increasing their half-life and penetration in the target cells [176]. Mesoporous silica NPs have been identified as a delivery system with great pharmacokinetic parameters, and they have been used to use photocaged-PROTAC (phoBET1) to degrade BRD4 in a controllable manner [177]. Nevertheless, it is still needed to assess in vivo the biodegradability and toxicity of this delivery system of PROTACs.

Polymeric NPs are made from natural or synthetic polymers with great biocompatibility properties. Nanohydrogels are cross-linked polymers with great capacity to hold water, and they are emerging as useful delivery systems due to their unique characteristics that make them biodegradable, biocompatible and stable [178]. These nanosystems have been applied to deliver compounds with different chemical nature like proteins, nucleic acids and SMIs [179], but its application with PROTAC molecules is less known and has shown difficulties in its encapsulation. Nevertheless, some studies have demonstrated its potentiality for targeted therapy. Delivery of NPs of DSPE-PEG and PDSA containing the ARV-771 PROTAC for targeted BRD4 degradation shown antitumor efficacy [180]. Moreover, a combined therapy using cRGD-modified nanoparticles (cRGD-P) co-delivering doxorubicin (DOX) and BRD4 PROTAC degrader ARV-825 showed synergistic tumor inhibition and a better efficacy than the individual administration of these compounds [181]. In the context of lung cancer, Guan et al. [182] developed a dual targeting and bio-responsive nano-PROTAC (RLDPB) using a pH and glutathione-responsive polymer (DS-PLGA) to load the PROTAC agent dBET6. The authors showed that RLDPB enhanced cellular uptake by lung cancer cells and accumulation in tumors due to its dual targeting structure, which includes cRGD and LLC cell membrane camouflage. This strategy significantly improved the therapeutic concentration of dBET6 in the tumor site. Also, the encapsulation of this nano-PROTAC increased their stability and prolonged their presence in the bloodstream. Additionally, responsiveness improved the release of dBET6 within the cells, ensuring a sustained therapeutic effect.

Recent advances have demonstrated the therapeutic potential of PROTACs in preclinical lung cancer models. ALK degraders such as dEALK1 and the linker-free Pro-BA achieved potent and selective antitumor activity in NSCLC xenografts, even overcoming resistance to kinase inhibitors [183,184]. In SCLC, a dual BCL-xL/BCL-2 PROTAC (753b) induced tumor regressions in NCI-H146 xenografts while avoiding dose-limiting thrombocytopenia, and an EZH2 PROTAC suppressed leptomeningeal metastases in chemoresistant SCLC mouse models [185,186]. In SMARCA4-deficient NSCLC, the VHL-based degrader A947 showed strong tissue penetration and sustained efficacy despite rapid plasma clearance, and next-generation orally bioavailable SMARCA2 degraders further validated this synthetic-lethal strategy in xenografts [187,188]. For EGFR-driven NSCLC, nanocarrier-assisted PROTAC delivery significantly enhanced in vivo antitumor efficacy in resistant xenografts, while co-delivery of EGFR and BRD4 degraders in a single nanoplatform achieved synergistic tumor suppression [189,190]. Collectively, these studies highlight diverse PROTAC strategies targeting oncogenic drivers or dependencies in NSCLC and SCLC, with robust evidence of efficacy across multiple animal models.

In recent decades, immunotherapy has made significant strides in cancer treatment, offering substantial benefits to certain cancer patients. Specifically, in the realm of lung cancer, immune checkpoint inhibitors have demonstrated considerable efficacy in treating NSCLC by blocking inhibitory pathways that suppress the immune response against cancer, thereby restoring and maintaining anti-tumor immunity. Clinically, PD-L1 and PD-1 blocking agents are widely employed, either alone or in combination with chemotherapy [191]. However, despite the notable success of T-cell-based immunotherapies in cancer treatment, they still encounter significant challenges such as severe side effects, low response rates, immunogenicity, and acquired resistance. Similarly, antibody-based immunotherapies face limitations like immunogenicity, as well as issues with membrane permeability, stability, and high production costs [192]. Some of these limitations have been overcome through the development of nano-PROTACs. As previously mentioned, these compounds, utilizing nanotechnology, can enhance drug concentrations at tumor sites. They can also be engineered to release drugs in response to light, temperature, or magnetic fields. Indeed, nano-PROTACs can be designed to provoke minimal off-target effects and offer improved biodistribution, membrane permeability, and controlled drug release. Additionally, based on their mechanism, they exhibit high selectivity and specificity compared to immunotherapy. Altogether, these advantages over immunotherapy highlight the potential of this targeted therapy.

### 3.4. ROS Inductor Nano PROTAC as a Potential Therapy for Lung Cancer

As tumors progress, the microenvironment becomes pro-oxidative. Therefore, tumoral cells require activating alterations, which promote oxidative response programs activation to survive, seeming to be crucial in the multi-step process of tumorigenesis, specifically in the acquisition of malignant and metastatic phenotypes. The effectiveness of actual targeted therapies against metastatic cancers has been disappointing, so it is essential to design useful treatments against malignant tumors, as they are the principal cause of mortality in patients with cancer. Driver mutations in *EGFR* are very common in lung cancer and, as has been previously described, there are currently targeted therapies that block the receptor and consequently the downstream pathways regulated by this RTK. Nevertheless, tumors tend to develop resistance to these therapies. Strikingly, tumor harboring these alterations in *EGFR* are commonly associated with pro-oxidant tumor microenvironment, opening a new window into the therapeutic armamentarium to use PROTAC against lung cancer.

The success of the strategy based on nanosystems for PROTAC delivery mainly depends on its optimal design and the ability to overcome the challenges faced by current lung cancer therapy: reduced therapeutic efficacy, multidrug resistance and secondary effects [193]. The small size of these nanosystems promotes the so-called enhanced permeability and retention (EPR) effect in tumors, increasing the effective drug concentration in the therapeutic target and reducing the required doses, among other advantages. It is also important to take into account other parameters in this design: (1) the specific anatomic-physiological characteristics of each tumor, essential to specifically concentrate and retain the nanosystem in the target of action, either using active or passive vehiculation strategies; and (2) the physicochemical stability conditions of the PROTAC molecule both during the synthesis and the administration of the nanosystem in the form of pressurized devices, nebulizer and/or dry powder device [194].

There is a wide variety of nanosystems (liposomes, exosomes, dendrimers, lipid and/or metallic nanoparticles, among others) that have demonstrated optimal therapeutic success in various types of lung cancer by delivering drugs of a chemical nature [185]. However, it should be taken into consideration that all these preclinical studies have been designed for the administration of such drugs by nebulization because of the limitation of using experimental animals, leaving aside dry powder and/or pressurized devices, which are more comfortable for the final patient. Furthermore, the fact that one of the most common routes of entry of nanosystems into the organism is the pulmonary route has made it possible to elucidate that the induction of ROS in this system is one of the toxic effects following the accumulation of nanomaterials, which would in turn serve as an alternative ROS-based therapy.

Considering the approximate size of PROTACs (10 nm according to [195]; 700–1100 Da according to [21] and their mentioned hydrophobicity, the nanosystems that could presumably exhibit better therapeutic results would be those offering a cavity of such size and hydrophobic nature (micells, liposomes, transferosomes, exosomes, dendrimers, nanohydrogels, etc.) in order to encapsulate the PROTAC and protect it from external degradation. The incorporation of specific ligands of target cell receptors would undoubtedly improve the selectivity of action of these nanosystems and reduce the side effects of current therapy, largely derived from the bystander effect. Finally, the materials controlling the vehiculation and release of PROTAC should ideally show compatibility with the cargo molecule and the tissues surrounding the target tissue. Also, as the system induces toxicity by activating ROS the objective of the ROS-inductor PROTAC to kill tumor cells would be enhanced.

Nanohydrogels based on polymers sensitive to external stimuli (pH, temperature, intra- and extracellular redox levels) such as those synthesized from N-isopropylacrylamide (NIPA), N-Hydroxyethyl acrylamide (HEAA) and N,N’-Cystaminebisacrylamide [196] are postulated as a successful therapeutic alternative. The ability to retain water without dissolving allows nanohydrogels to mimic tissues and adhere to the respiratory mucosa and epithelium, increasing the retention of these systems in the respiratory tract. Moreover, the presence of an acidic medium (characteristic of the tumor microenvironment) triggers the transition from the collapsed to the swollen state, favoring the diffusion of the physiological medium into the system and promoting the internal erosion mechanism from the oxidation of the disulfide bridges of the crosslinking agent, which leads to the biodegradation of these nanosystems and to the delayed release of PROTAC. Such a latent release phase would protect the PROTAC from premature external enzymatic and chemical degradation. Also, the nanometer size of these systems, as occurs in other nanomaterials, increases the surface/volume ratio of the starting PROTAC. This surface area is available to incorporate ligands that will bind specifically to the receptors overexpressed in the tumors to be treated.

**Figure 4:**
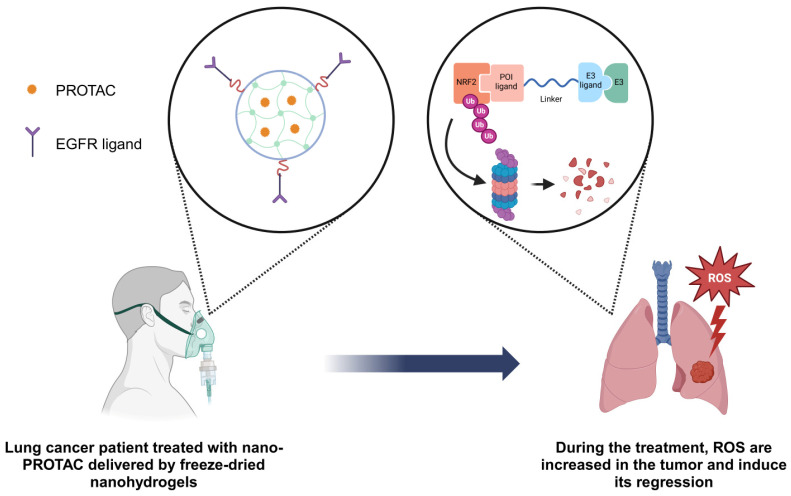
ROS inductor nano-PROTAC delivered by freeze-dried nanohydrogels for lung cancer patients. The proposed therapy is useful for EGFR drug-resistant lung tumors with wild-type NRF2 using nano-PROTAC delivered by EGFR-targeting nanohydrogels. This strategy aims to degrade NRF2, sensitizing tumors to ROS-induced cell death and promoting tumor regression.

Considering all the previous evidence of the ROS effect in tumors and the last advances in nanotechnology, we would like to propose a potential therapy for drug-EGFR resistant lung tumors with wild-type (WT) *NRF2* genotype based on the delivery of nano-PROTAC with freeze-dried nanohydrogels (Figure 4). Nanohydrogels would be characterized by having EGFR ligands to optimize the vehicle delivery specifically in the tumors harboring EGFR overexpression. The PROTAC molecule required for the treatment would be designed to have a NRF2-binding element and a CRBN ligand, whose efficacy in NRF2 degradation has been previously demonstrated in NSCLC models [197]. Moreover, ROS cell-death induction in LUSC patient-derived organoids and xenografts has been demonstrated to be successful when NRF2 is inhibited in tumors with no alterations in this pathway [17]. Thus, the degradation of NRF2, the pivotal guardian of cellular oxidative stress, would render tumor cells defenseless against the harsh oxidative microenvironment of the tumor, ultimately driving them toward cell death and tumor regression.

While nano-PROTACs offer numerous benefits, their delivery systems require optimization to address the limitations observed in clinical applications. Key challenges in this research area include the absence of dependable methods for analyzing the structure of nano-PROTAC assemblies, instability in the polymer-to-drug ratio, potential particle aggregation in vivo, issues with long-term storage stability, and high production costs, among others [170,198]. Current research on polymer nanoparticle-based PROTAC drugs is particularly challenged by the need to efficiently encapsulate PROTACs, create intelligent delivery systems, and achieve successful clinical translation due to high variability in mass production, among other issues. Furthermore, the safety profiles of many nano-PROTAC components have not been thoroughly assessed and require further study. Additionally, concerns about toxicity from unexpected immunogenic reactions to polymers and their excipients, lack of selectivity leading to off-target effects, suboptimal therapeutic outcomes, and increased systemic toxicity may complicate their use [198]. For example, the frequent use of polyethylene glycol (PEG) to anchor a ligand to the nano-system surface can cause pseudo-allergic reactions due to complement activation. This potential off-target effect is being addressed through a combination of strategies, such as photodynamic therapy and chemical modifications of targeted groups [170]. Therefore, developing new evaluation methods and pharmacological systems to facilitate and adapt to the widespread clinical application of a new paradigm of nano-PROTACs is necessary to ensure their significant clinical benefits in the future.

As was mentioned before, PROTACs have exhibited low water solubility, poor cellular permeability, off-target toxicity, and hook effects, which together limit their effectiveness and clinical use. Additionally, the main administration routes of PROTACs, parenteral and oral administration, show fast renal clearance, requiring frequent dosing and potentially complicating their pharmacokinetic profiles, leading to reduced bioavailability. To overcome the challenges associated with both parenteral and oral administration of PROTACs, it is crucial to explore alternative routes, such as inhalation therapy. PROTACs need to reach the cytosol of target cells to interact with their POI and trigger proteasomal degradation. Nano-system-based drug delivery systems offer a promising solution to these inherent issues by enhancing the physicochemical properties of PROTACs. These nano-systems can be engineered to release PROTACs in a controlled way, ensuring sustained delivery and continuous degradation of target proteins over time. However, current in vivo preclinical studies of PROTAC-loaded nano-system formulations are still not enough to confirm the efficacy by these formulations and further evaluation in animal models is necessary to fully characterize the drug delivery profile of all types of existing nano-systems [198]. On the other hand, the development of nano-PROTAC-based therapies encounters substantial obstacles concerning scalability and regulatory requirements. A major challenge in nanomedicine product development is the shift from small-scale laboratory batches to large-scale industrial production, along with the selection of excipients necessary for manufacturing high-quality pharmaceuticals [199]. Additionally, the regulatory guidance of nanomedicine remains unclear and unconsolidated, which hinders the control and adaptation of manufacturing processes and production scale-up [200].

To address these challenges, the clinical translation of nano-systems requires specific protocols, stringent criteria for the characterization of materials and biological mechanisms, and the development of new technology platforms. Additionally, better clinical in vitro models that replicate real conditions and standardized statistical analyses are essential to obtain marketing authorization for innovative technologies designed to meet global healthcare and treatment needs.

## 4. Concluding Remarks

Lung cancer remains a leading cause of mortality worldwide, characterized by poor prognosis and low survival rates. This trend is largely driven by increased tobacco use, industrialization, and air pollution. Given these statistics, there is an urgent need for effective therapeutic strategies. Despite advancements in diagnostics and treatments, lung cancer still lacks highly effective treatments. Exploring reactive oxygen species (ROS), which play a dual role in cancer development by both promoting and inhibiting tumorigenesis, offers a promising framework for developing new drugs to target lung cancer cells. ROS regulate key signaling pathways such as MAPK, PI3K/Akt, and NF-κB, which are crucial in cancer initiation, progression, and metastasis. These oxidant species can also activate cell death pathways, presenting a unique opportunity for targeted therapies. Research indicates that wild-type NRF2/KEAP1 lung tumors, which account for approximately 60% of lung malignancies, are sensitive to ROS induction. Additionally, mutated EGFR1 lung tumors tend to exhibit high ROS levels. These findings highlight the critical role of ROS in developing targeted lung cancer therapies.

In recent decades, proteolysis-targeting chimeras (PROTACs) have emerged as a promising alternative to small molecule inhibitors (SMIs) for cancer treatment, addressing limitations such as undruggability, off-target effects, and drug resistance. Despite their potential, PROTACs face challenges like limited cell penetration and potential toxic side effects. Nanotechnology has introduced “nano-PROTACs,” which enhance tissue accumulation, membrane permeability, and controlled release.

Based on the evidence of ROS effects in tumors and advancements in nanotechnology, here a potential targeted therapy is proposed for drug-EGFR resistant lung tumors with wild-type NRF2 genotype. This therapy involves delivering nano-PROTAC using freeze-dried nanohydrogels, which are optimized with EGFR ligands for targeted delivery to tumors with EGFR overexpression. The PROTAC molecule would include an NRF2-binding element and a CRBN ligand, effective in NRF2 degradation. Inhibiting NRF2 in tumors without pathway alterations has shown success in inducing ROS cell death. Therefore, degrading NRF2 would leave tumor cells vulnerable to oxidative stress, leading to cell death and tumor regression.

## Data Availability

No new data were created or analyzed in this study.
